# Dietary-Induced Low-Grade Inflammation in the Liver

**DOI:** 10.3390/biomedicines8120587

**Published:** 2020-12-09

**Authors:** Nicole Power Guerra, Luisa Müller, Kristin Pilz, Annika Glatzel, Daniel Jenderny, Deborah Janowitz, Brigitte Vollmar, Angela Kuhla

**Affiliations:** 1 Rudolf-Zenker-Institute for Experimental Surgery, Medical University Rostock, 18057 Rostock, Germany; nicole.guerra@uni-rostock.de (N.P.G.); luisa.mueller2@uni-rostock.de (L.M.); annika.glatzel@uni-rostock.de (A.G.); daniel.jenderny@uni-rostock.de (D.J.); brigitte.vollmar@med.uni-rostock.de (B.V.); 2 Department of Psychosomatic Medicine and Psychotherapy, University of Rostock, 18147 Rostock, Germany; 3 Department of Psychiatry, University of Greifswald, 17489 Greifswald, Germany; kristin.pilz@med.uni-greifswald.de (K.P.); deborah.janowitz@med.uni-greifswald.de (D.J.)

**Keywords:** high fat diet, control diet, non-alcoholic fatty liver disease, liver inflammation, low-grade inflammation

## Abstract

The literature describes a close correlation between metabolic disorders and abnormal immune responses, like low-grade inflammation (LGI), which may be one mechanistic link between obesity and various comorbidities, including non-alcoholic fatty liver disease (NAFLD). In our study, we investigated the influence of dietary composition on obesity-derived LGI in the liver. We used a dietary induced obesity mouse model of C57BL/6J mice fed with high fat diet (HFD, 60% fat, 20% protein, 20% carbohydrates) and two different controls. One was rich in carbohydrates (10% fat, 20% protein, 70% carbohydrates), further referred to as the control diet (CD), and the other one is referred to as the standard diet (SD), with a more balanced macronutrient content (9% fat, 33% protein, 58% carbohydrates). Our results showed a significant increased NAFLD activity score in HFD compared to both controls, but livers of the CD group also differed in their macroscopic appearance from healthy livers. Hepatic fat content showed significantly elevated cholesterol concentrations in the CD group. Histologic analysis of the cellular immune response in the liver showed no difference between HFD and CD and expression analysis of immunologic mediators like interleukin (IL)-1β, IL-6, IL-10 and tumor necrosis factor alpha also point towards a pro-inflammatory response to CD, comparable to LGI in HFD. Therefore, when studying diet-induced obesity with a focus on inflammatory processes, we encourage researchers to carefully select controls and not use a control diet disproportionally rich in carbohydrates.

## 1. Introduction

Abnormal and excessive accumulation of adipose tissue in the context of severe overweight and obesity is one of the most challenging diseases of the 21st century. This is primarily due to the steadily increasing number of obese patients who are getting younger and younger [1]. In addition to a wide variety of cultural and social influences and a lack of exercise, a permanent oversupply of food rich in calories, and mostly also in fat, leads to weight gain with serious health issues.

As a consequence, obesity is one of the leading causes of the metabolic syndrome, which was described in 1989 by Kaplan [2]. The metabolic syndrome is associated with many other diseases [3,4] including non-alcoholic fatty liver disease (NAFLD), which is seen as the hepatic manifestation of it [5]. One assumed reason for the prevalence of NAFLD and many other concomitant and secondary diseases of obesity is the persistence of systemic low-grade inflammation (LGI), starting from adipose tissue. However, there is no strict definition of LGI. In general, dietary-induced LGI is a sterile inflammation, which has also been given the name “metaflammation” (an inflammation of metabolic tissue) [3], which is highly entwined with immunometabolism [6]. Since the LGI differs in its triggering mechanisms from an infectious inflammation, both also likely differ in their consequences. While infectious inflammation in its physiological function triggers an immune response of the organism, sterile inflammation has predominantly pathological consequences, e.g., via the alteration of homeostatic checkpoints and the development of autoinflammatory disorders [7].

Interestingly, a close correlation between metabolic diseases and abnormal immune responses such as LGI is observed [3,8]. White adipose tissue is capable of expressing both, metabolic and immunological mediators [9], whose effects are not only local but can affect other organs like the liver or have a systemic impact. This is also shown by a generalized moderate upregulation of pro-inflammatory signaling cascades in overweight and obesity. Important mediators in this context include interleukin (IL)-1β, IL-6 and tumor necrosis factor alpha (TNFα) [10,11,12]. Moreover, a downregulation of anti-inflammatory mediators such as IL-10 has been reported [12]. All of the above mentioned mediators take part in well-orchestrated and tightly regulated signaling cascades and derailments of them may be responsible for a LGI-mediated interaction between obesity and NAFLD [13,14]. However, which long-term influences in the diet are present, defined as at least 6 months of corresponding diet, on obesity-derived LGI in the liver has not yet been investigated in detail in mice.

A diet-induced obesity mouse model consisting of a 60% high fat diet (HFD) is used in this project to observe the effects of obesity-derived LGI on the liver. As an additional approach, we wanted to investigate the impact of dietary composition of two different low-fat controls on the liver, with a special focus on LGI. One control group received the HFD-manufacturers recommended control diet (CD), broadly used in research [15,16,17,18]. This CD matches the HFD regarding sucrose content (as a percent of calories) and fiber structure but has generally a high carbohydrate content and in particular a high starch content ([19]; Product Data—DIO Series Diets, Research Diets Inc., Lane, NJ, USA). As the literature hints towards the role of carbohydrate-rich diets as promotors of systemic LGI, e.g., by oxidative stress induction [20,21], it would be of high interest to have a closer look if this mechanism affects the liver as well. In contrast, the literature suggests that protein-rich diets have anti-inflammatory effects and also reduce liver fat [22], which is an important note when considering studying dietary-induced effects of LGI on the liver. Therefore, the other control group received the in-house standard diet (SD) with matched calorie content to CD but lower carbohydrate content and increased protein content compared to CD and HFD.

## 2. Experimental Section

### 2.1. Animal Models

**Figure 1:**
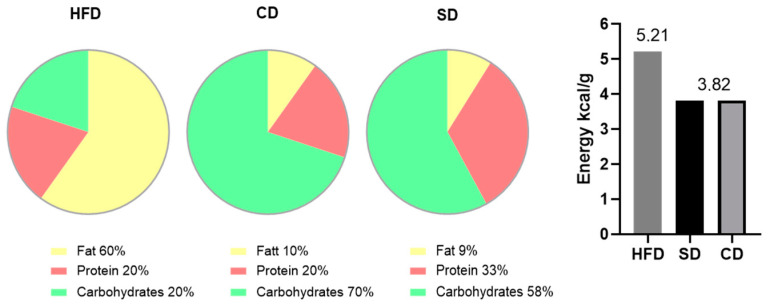
Composition of high fat diet (HFD), control diet (CD) and standard diet (SD) in % of total calories and energy density in kcal/g.

For the experiments, female C57BL/6J mice at the age of 4 weeks were purchased from Charles River (Sulzfeld, Germany). In compliance with our own previous and ongoing investigations, female mice were used for comparability between different studies. Mice were kept in standard cages with 4 to 5 animals per cage, in a temperature controlled room (21 ± 3 °C) with a 12/12 h day-night cycle (lights on from 06:00 am to 06:00 pm CET) containing a twilight period of 30 minutes. The mice were blindly divided into three groups, which were fed different diets and water supply ad libitum over a period of 6 months. After one week of acclimatization, the food was adjusted to the corresponding diet, with designated compositions shown in Figure 1. One group received an HFD (D12492; Research Diets Inc., Lane, NJ, USA), hereinafter referred to as the HFD group (*n* = 31). The other group received the recommended CD (D12450J; Research Diets Inc., Lane, NJ, USA), hereinafter referred to as the CD group (*n* = 16). The third group received SD (ssniff^®^ R/M-H, ssniff Spezialdiäten GmbH, Soest, Germany) and is hereinafter referred to as the SD group (*n* = 15).

All animal experimental work was carried out with permission of the local Animal Research Committee (Landesamt für Landwirtschaft, Lebensmittelsicherheit und Fischerei (LALLF)) of the state Mecklenburg-Western Pomerania (LALLF M-V/TSD/7221.3-2-001/18, approved on 1 March 2018) and all animals received human care according to the EU Directive 2010/63/EU.

### 2.2. Blood Sampling and Tissue Preparation

Mice were anaesthetized with 5 vol.% isoflurane (Baxter, Unterschleißheim, Germany), 0.8 L/min O_2_ (Air Liquide, Hamburg, Germany) and 1.25 L/min N_2_O (Air Liquide, Hamburg, Germany) and blood was taken retrobulbary to exsanguinate the mice. Blood samples were kept at 4 °C until plasma preparation the same day. Therefore, samples were centrifuged at 1200 rpm and 6 °C for 10 min (Centrifuge 5424, Eppendorf, Leipzig, Germany) and supernatant was collected and stored at −80 °C. Then, mice were transcardially perfused with 20–25 mL 0.9% NaCl (Braun, Melsungen Germany) with an estimated flow rate of 2.28–2.83 mL/min. For histological and immunohistochemical analysis, the left lateral liver lobe was dissected and fixed in 4% paraformaldehyde (PFA, ChemCruz, Dallas, TX, USA) solution for five days, embedded in paraffin (Carl Roth, Karlsruhe, Germany) and sectioned in 4 µm thin tissue slices. For molecular analysis, the remaining liver was homogenized and snap frozen in liquid nitrogen and stored at −80 °C.

### 2.3. Biochemistry

Directly after blood collection, blood sugar concentration in the naive blood sample was assessed with the glucose meter Contour^®^XT (Bayer, Leverkusen, Germany) according to the manufacturer´s instructions. In the stored plasma samples, aspartate aminotransferase (AST) and alanine aminotransferase (ALT) activities were measured spectrophotometrically as indicators of hepatocellular disintegration and necrosis. The extinction at 340/378 nm was measured with the cobas®c111Analyzer (Roche Diagnostics GmbH, Penzberg, Germany). Measurement of plasma triglycerides was performed using Triglyceride Colorimetric Assay Kit (Nr.: 10010303, Cayman Chemical Company, Hamburg, Germany) according to the manufacturer’s instructions. Results are provided in the Supplementary Materials (Table S1).

### 2.4. Histology, Immunohistochemistry and Image Analysis

Hematoxylin (Merck, Darmstadt, Germany) and eosin (Merck, Darmstadt, Germany) (H&E) staining was performed using standard protocols. Pictures were recorded on a microscope type BX51 with a Color View Soft Imaging System and the corresponding software cellSens Standard 1.14 (all from Olympus, Hamburg, Germany).

From the H&E stained specimens, analyses of tissue content of microvesicular fat was performed using the public domain image analysis software ImageJ (v.1.47) (protocol provided in the supplements as ImageJ Code S1). Furthermore, a NAFLD Activity Score (NAS) was generated to characterize diet-induced liver damage. Following the description by Kleiner et al. [23], the parameters steatosis (score 0–3), hepatocellular ballooning (score 0–2) and lobular inflammation (score 0–3) were used to calculate NAS (total score 0–8). Steatosis was assessed at 50× magnification and ballooning at 100× magnification. Inflammation was assessed by counting inflammatory foci from 20 representative low-power fields (LPF) (200× magnification) with an inflammatory focus characterized as a grouping of at least five inflammatory cells in the tissue, which are not arranged in a row [24]. Examples for the different assigned scores are provided as representative images in the supplements (Figure S1).

For assessment of tissue infiltration of granulocytes as another hallmark of manifestation of LGI in the liver, sectioned paraffin-embedded liver tissue was stained for chloracetate esterase (CAE) with Naphthol AS-D chloroacetate (Sigma-Aldrich, Darmstadt, Germany) and counterstained with hematoxylin (Merck, Darmstadt, Germany). For quantification, the total number of hepatocytes and CAE positive cells (CAE^+^) was counted in 20 consecutive high-power fields (HPF) at 400× magnification.

As a second cellular indicator for LGI in the liver, macrophages were stained immunohistochemically. Therefore, overnight incubation (4 °C) with the first antibody (rat anti mouse-F4/80 [MCA497] from Bio-Rad, Hercules, CA, USA) was followed by 1 h incubation at room temperature with the secondary antibody (goat anti rat [MCA497] from Bio-Rad, Hercules, CA, USA) stained with the chromogen Permanent Red (Ref. K0640, DAKO GmbH, Jena, Germany) and counterstained with hematoxylin (Merck, Darmstadt, Germany). For quantification, the total number of hepatocytes was counted in 20 consecutive HPF at 400× magnification and semiautomatic quantification of F4/80 positive cells (F4/80^+^) was performed via ImageJ (protocol provided in the supplements as ImageJ Code S2).

### 2.5. Cholesterol Assay

For assessment of hepatic cholesterol content, Cholesterol Quantitation Kit (Calbiochem^®^, Merck, Darmstadt, Germany) was performed according to manufacturer instructions from 30 µg snap frozen liver tissue.

### 2.6. Quantitative Real-Time PCR

RNA isolation from snap frozen liver tissue was performed with RNeasy Mini Kit (Qiagen, Venlo, The Netherlands) according to the manufacturer’s instructions. RNA integrity was verified by agarose gel electrophoresis and RNA concentration was assessed by absorption measurement with NanoDrop (Thermo Fisher Scientific, Waltham MA, USA). Isolated RNA was transcribed into cDNA with SuperScript™ (Invitrogen, Thermo Fisher Scientific, Waltham MA, USA) and deoxyribonucleosidtriphosphates (Thermo Fisher Scientific, Waltham, MA, USA) were added. Cytokine analyses were performed via quantitative real-time PCR in a BioRad iQ5 Multicolor Real Time PCR Detection System (Conquer Scientific, San Diego, CA, USA) with iQ™ SYBR^®^ Green Supermix (Bio-Rad, Hercules, CA, USA). Primer sequences are shown in Table 1. Measurement results are corrected against the housekeeping gene 40S ribosomal protein S18 (RPS18) and relative quantification was carried out by usage of the 2^−ΔΔCT^ method.

### 2.7. Statistical Analysis

Statistical analysis was performed using GraphPad Prism 8.0.1 (GraphPad Software Inc., San Diego, CA, USA). Data were checked for normality with the Kolmogorov–Smirnov test (for scoring data) or Shapiro–Wilk test and variances of ANOVA were verified by Bartlett’s test. If SDs were not significantly different with *p* > 0.05, an ordinary one-way ANOVA was performed followed by Turkey post hoc test, otherwise Brown–Forsythe and Welch ANOVA followed by Tamhane’s T2 multiple comparisons test was performed. If data were not normally distributed, the Kruskal–Wallis test with Dunn’s post hoc test for multiple comparisons was conducted. Data are presented as mean ± standard deviation and statistical significance was set at *p* < 0.05. The ROUT method based on the false discovery rate (Q = 0.01) was used to identify and remove outliers if possible and necessary. For further details, see figure legends.

## 3. Results

### 3.1. Dietary Impact on Body and Liver Weight

**Figure 2:**
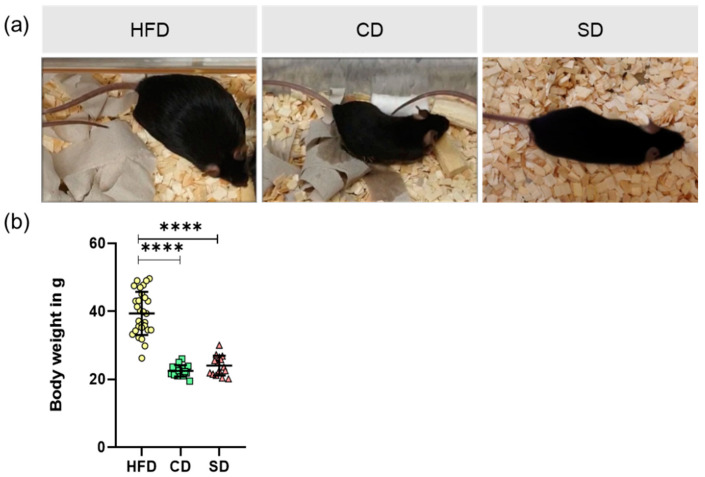
(**a**) Representative images of appearances from mice fed high fat diet (HFD), control diet (CD) or standard diet (SD); (**b**) Body weights of mice in the different groups (HFD: *n* = 29; CD: *n* = 15, SD: *n* = 15), presented as mean ± standard deviation. Significance of differences between the groups was tested by Brown–Forsythe and Welch ANOVA, **** *p* < 0.0001.

After feeding the mice their respective diet for 6 months, their body weight was measured and liver tissue was collected for further analysis. Animals are shown as representative images (Figure 2a). Analysis of body weight revealed significantly elevated values in the HFD group (Figure 2b, *p* < 0.0001 vs. CD and SD group). Body weight did not differ between the CD and SD group (Figure 2b).

**Figure 3:**
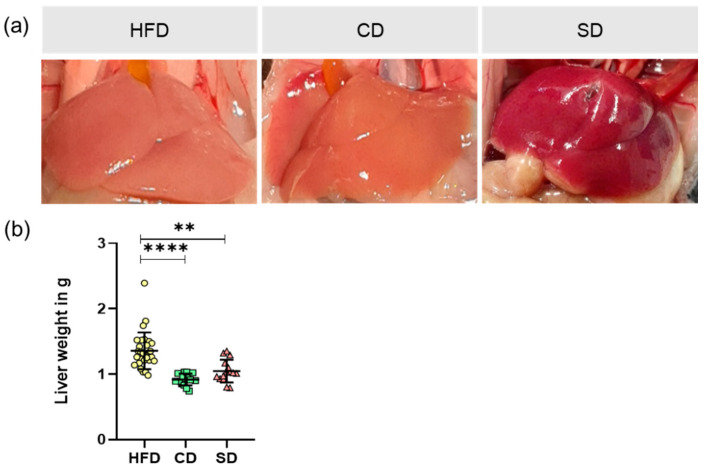
(**a**) Representative in situ images of mice livers from mice fed high fat diet (HFD), control diet (CD) or standard diet (SD); (**b**) Liver weight of the different groups (HFD: *n* = 29; CD: *n* = 15, SD: *n* = 15) presented as mean ± standard deviation. Significance of differences between the groups was tested by one-way ANOVA on Ranks (Kruskal–Wallis); **** *p* < 0.0001, ** *p* < 0.01.

Macroscopic appearances of the livers in situ are shown as representative images (Figure 3a). While dissecting the liver, we noticed a visual deviation of both the HFD and CD group to a healthy looking liver as displayed by the SD group. Analysis of liver weight again revealed significantly elevated values in the HFD group (Figure 3b) (*p* < 0.0001 vs. CD group and *p* = 0.013 vs. SD group) and no significant difference between the CD and SD group.

### 3.2. Dietary Induced Liver Steatosis

**Figure 4:**
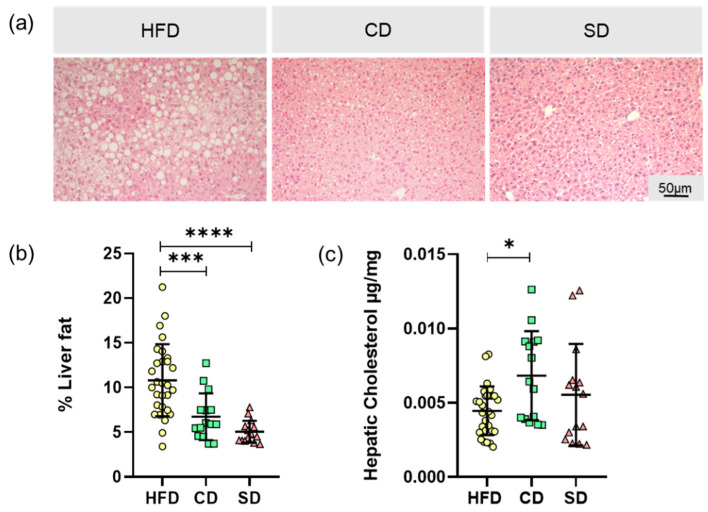
(**a**) Representative LPFs of the livers from mice fed high fat diet (HFD), control diet (CD) or standard diet (SD) (200× magnification, scale bar representing 50 µm valid for all three images); (**b**) Percentage of hepatic vesicular fat content; (**c**) Hepatic cholesterol concentration of the HFD, CD and SD group. Data (HFD: *n* = 29; CD: *n* = 15, SD: *n* = 15) presented as mean ± standard deviation. Significance of differences between the groups was tested by Brown–Forsythe and Welch ANOVA in (**b**) or one-way ANOVA on Ranks (Kruskal–Wallis) in (**c**); **** *p* < 0.0001, *** *p* < 0.001, * *p* < 0.05.

The basis for the increased liver weight is probably diet-induced liver steatosis. To analyze this parameter, the microvesicular liver fat content was determined in H&E stained tissue, with representative images shown in Figure 4a. Liver tissue of the HFD group showed excessive macro- and microvesicular fat deposits. In addition, in liver tissue of the CD group, fat depots, mostly microvesicular, were found. Image analysis led to a significantly higher fat quantity in HFD compared to the CD and SD group (Figure 4b, *p* = 0.0007 vs. CD group and *p* < 0.0001 vs. SD group) but not between the SD and CD group. Tissue fat content alone therefore did not serve as an explanation for the macroscopically observed brightening of livers of the CD group. Subsequently we found that in the livers of the CD group, the amount of hepatic cholesterol was significantly higher than in the HFD group (*p* = 0.024) but not in the SD group (Figure 4c).

**Figure 5:**
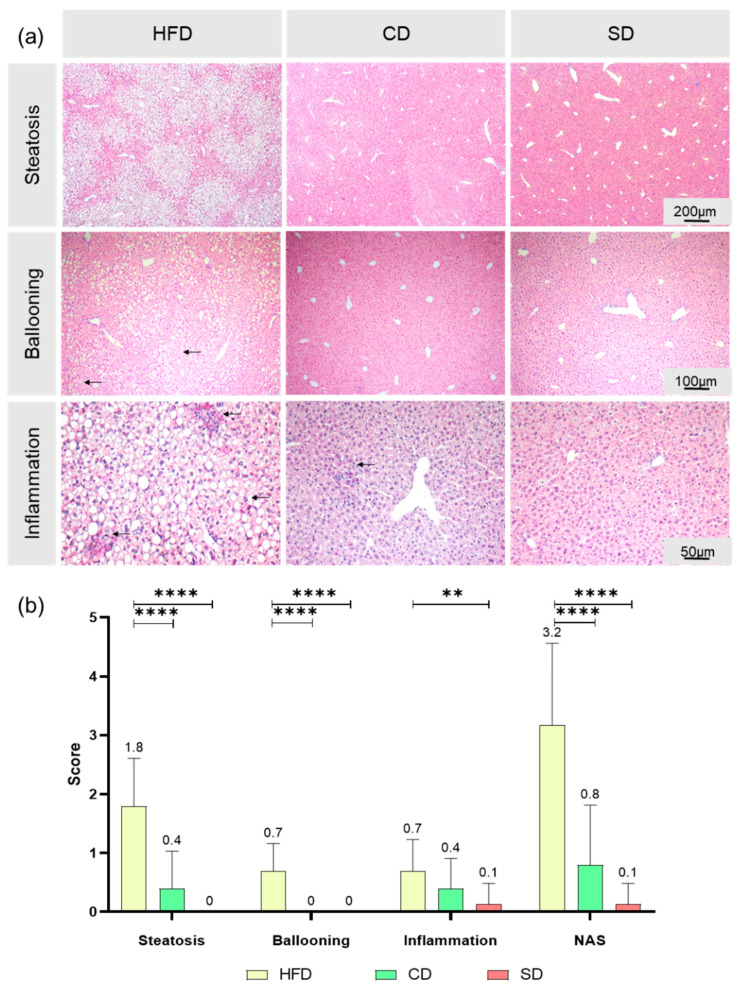
(**a**) Representative images of steatosis (50× magnification, scale bar represents 200 µm valid for all three images), ballooning (100× magnification, scale bar representing 100 µm valid for all three images) with black arrows indicating damaged cells, and inflammation (200× magnification, scale bar representing 50 µm valid for all three images) with black arrows indicating inflammatory foci in livers from mice fed high fat diet (HFD), control diet (CD) or standard diet (SD); (**b**) Assessments of scores for steatosis, ballooning and inflammation as well as calculation of NAS for the groups (HFD: *n* = 29; CD: *n* = 15, SD: *n* = 15). Data presented as mean ± standard deviation. Significance of differences between the groups was tested by one-way ANOVA on Ranks (Kruskal–Wallis); **** *p* < 0.0001, ** *p* < 0.01.

As an additional parameter to assess the impact of the different diets on the liver, we calculated the NAS for the different groups, according to exemplary scoring in Supplement Figure S1. Fat deposits (representative images shown in Figure 5a) in the HFD group indicated pathological changes in all liver samples. The HFD group, with mostly a score of 2, showed a significantly higher liver steatosis than the CD and SD group (Figure 5b), both *p* < 0.0001) with a score mostly between 0 and 1. The CD group did not differ significantly from the SD group, which is in line with the above shown quantification of liver fat. The SD group constantly showed a score of 0 in all samples, which corresponds to a healthy liver. As a second parameter for the NAS, ballooning, a form of cell injury and death through fat accumulation, was assessed (Figure 5a). In all our samples, none of the hepatocytes showed ballooning injury to any extent, therefore score 2 was not assigned once. As some samples showed a few ballooned hepatocytes in the HFD group, represented by a score of 1, the scoring was significantly higher than in the CD and SD groups (Figure 5b), *p* < 0.0001) as there were no ballooned cells observed in the livers of the low-fat diets, assigned to score 0. The third parameter to calculate NAS is lobular inflammation (Figure 5a). In all samples, no massive inflammation of liver tissue, assigned to score 3, was found. With 2–4 inflammatory foci per LPF, a stout inflammation, defined as score 2, could be seen in a few livers of the HFD group. Interestingly, when it comes to inflammation, there was only a significant difference between the HFD and SD group (Figure 5b), *p* = 0.003) but not between the HFD and CD group. In the overall result, the examined parameters steatosis, ballooning and inflammation contributed to the NAS result with significantly higher values in the HFD group compared to the CD and SD group (Figure 5b), *p* < 0.0001 vs. CD and SD group). This hints toward a damaging effect of the HFD on the liver.

### 3.3. Dietary-Induced LGI in the Livers of the HFD Group and CD Group

**Figure 6:**
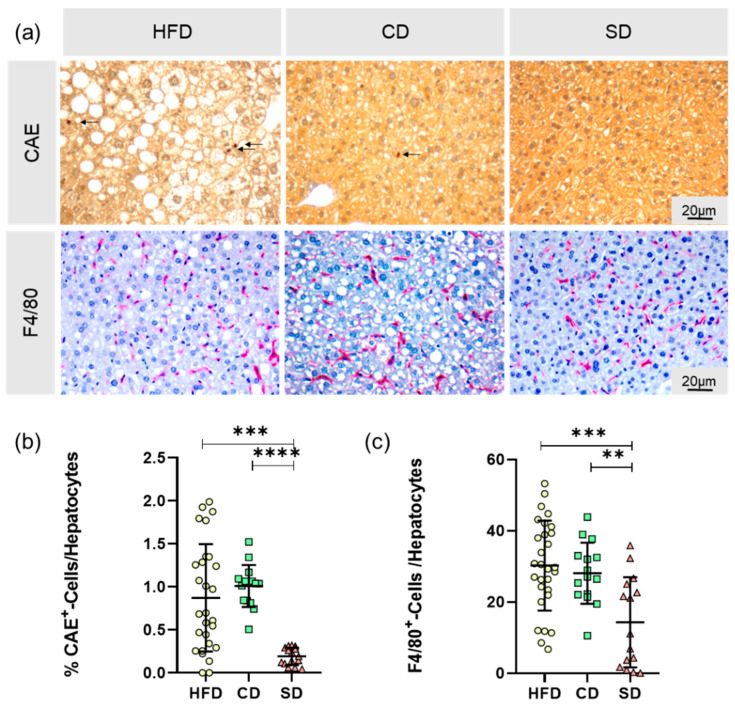
(**a**) Representative images of CAE-staining with CAE^+^-cells indicated by arrows and F4/80-staining with F4/80^+^-cells stained in red (both at 400× magnification, scale bar representing 20 µm valid for all images) of livers from mice fed high fat diet (HFD), control diet (CD) or standard diet (SD); (**b**) Relative amount of granulocytes (CAE^+^) (HFD: *n* = 28; CD: *n* = 15, SD: *n* = 15); (**c**) Relative amount of macrophages (F4/80^+^) (HFD: *n* = 29; CD: *n* = 15, SD: *n* = 15). Data presented as mean ± standard deviation. Significance of differences between the groups was tested by one-way ANOVA on Ranks (Kruskal–Wallis) in (**b**) or ordinary one-way ANOVA in (**c**); **** *p* < 0.0001, *** *p* < 0.001 ** *p* < 0.01.

As already indicated by macroscopy changes in the livers of the CD group, potential pathogenic effects of the carbohydrate rich diet emerged, and only a significant difference between the HFD and SD group was observed when scoring lobular inflammation in the NAS assessment. Thus, we chose to gain a more in-depth look into LGI processes in the liver. Therefore, we analyzed the cellular immune reaction by quantification of CAE^+^ and F4/80^+^ cells in the liver, relativized to the total number of hepatocytes per HPF (Figure 6a). The data we obtained substantiate the results from inflammation scoring in NAS, again showing a significant difference between the HFD and SD group (Figure 6b), CAE^+^: *p* = 0.0003, Figure 6c), F4/80^+^: *p* = 0.0002,) but not between the HFD and CD group. Additionally, in both analyses, there was a significant difference between both controls (Figure 6b), CAE^+^: *p* < 0.0001, Figure 6c), F4/80^+^: *p* = 0.006). In conclusion, compared to the SD group, the CD group had significantly increased amounts of immune cells in the liver.

**Figure 7:**
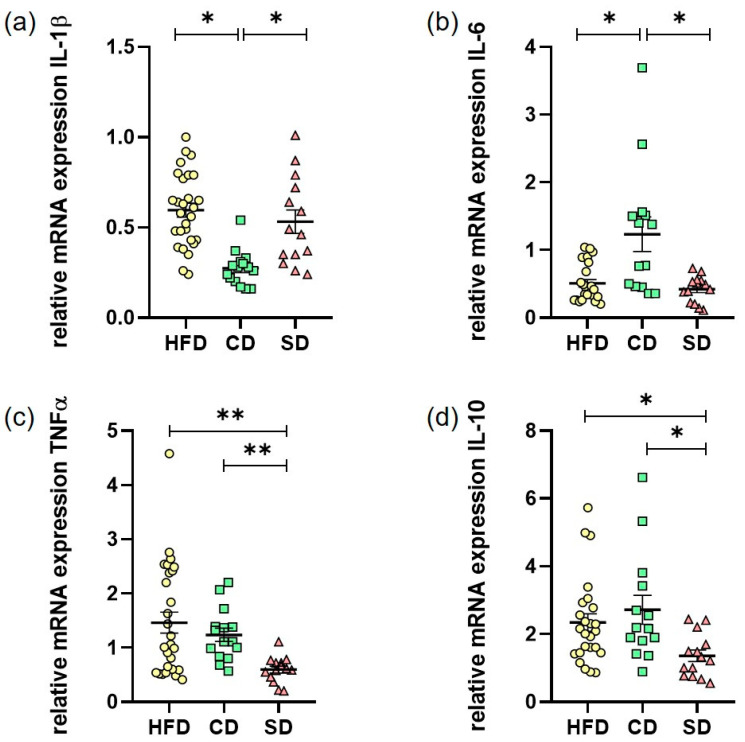
(**a**) Hepatic mRNA expression in the high fat diet (HFD), control diet (CD) or standard diet (SD) group of IL-1β (HFD: *n* = 28, CD: *n* = 14, SD: *n* = 14); (**b**) Hepatic mRNA expression of IL-6 (HFD: *n* = 24, CD: *n* = 15, SD: *n* = 14); (**c**) Hepatic mRNA expression of TNFα (HFD: *n* = 28, CD: *n* = 15, SD: *n* = 14); (**d**) Hepatic mRNA expression of IL-10 (HFD: *n* = 25, CD: *n* = 15, SD: *n* = 14). Data presented as 2^−ΔΔCt^ values determined by quantitative real-time PCR; Data presented as mean ± standard deviation. Significance of differences between the groups was tested by ordinary one-way ANOVA in (a) or one-way ANOVA on Ranks (Kruskal–Wallis) in (**b**–**d**) or; ** *p* < 0.01, * *p* < 0.05.

In addition to the cellular immune response, we investigated the humoral immune response in liver LGI. As one of the most important synthesis organs, the liver is able to produce many immunogenic mediators. For quantification of the pro-inflammatory cytokines IL-1β, IL-6 and TNFα as well as the anti-inflammatory IL-10, hepatic RNA expression was evaluated by quantitative real-time PCR (Figure 7a–d)). The IL-1β levels in the CD group were significantly lower than in the HFD (*p* < 0.0001) and SD group (Figure 7a), *p* < 0.0001 vs. HFD group, *p* = 0.0021 vs. SD group). This was astonishingly reverted for IL-6 levels where we saw a significant increase in comparison to the HFD and SD group (Figure 7b), *p* = 0.0106 CD vs. HFD group, *p* = 0.0124 CD vs. SD group). Only TNFα values were as expected highest in the HFD group, which was significant compared to SD (Figure 7c), *p* = 0.0052) but not to the CD group. In addition, a significant elevation of TNFα expression in the CD group compared to the SD group (Figure 7c), *p* = 0.005) was found. For IL-10, we found significantly increased expression when comparing the HFD and CD group with the SD group (Figure 7d), *p* = 0.0136 HFD vs. SD and *p* = 0.0275 CD vs. SD). The results depicting overall a heterogeneous effect of LGI on cytokine expression, but of note, the CD and SD group always differed significantly.

## 4. Discussion

Obesity is not merely a health risk itself, but often associated with other concomitant and secondary diseases [3,4,25]. It has become a serious disease of the 21st century due to its steadily increasing prevalence [1], and therefore is of great interest to study underlying disease mechanisms. One mechanism believed to contribute to disease progression is dietary-induced LGI starting from adipose tissue and later also becoming systemic and manifesting in other organs like the liver [26]. This is explainable by the close phylogenetic relationship between the liver and adipose tissue as well as the immune and hematopoietic system, which have evolved from formerly common structures [3]. In humans, as well as in many other mammals, hepatocytes and adipocytes are in close proximity to immune cells and have unhindered access to blood vessels [27,28,29]. Assuming that within the framework of the common evolutionary lineage, common signaling molecules and pathways have also been conserved.

To draw more detailed conclusions of dietary effects on liver LGI, a diet-induced obesity mouse model was used in this study. We chose a HFD model to map the development of obesity and especially inflammation. Due to a diet rich in calories and fat, C57BL/6J mice on HFD show a diet-induced obesity phenotype mirroring the situation in obese humans in western industrial countries [30]. The induction of obesity by such a model is usually less artificial and therefore findings are better translatable to humans. This is especially the case because corresponding genotypes of genetically obese mice models like Lep^ob^ /Lep^ob^ mice or mice with tubby gene mutations [31,32] are rarely found in humans.

In our work, we aimed to investigate the dietary impact on effects of obesity-derived LGI in the liver. With our experimental approach, we were able to show an adverse effect of HFD on the liver, represented mainly through an elevated NAS. Surprisingly, when looking especially on LGI processes, the carbohydrate-rich control diet also exacerbated the pro-inflammatory response in the liver.

In terms of assessing dietary effects on mice, body and liver weights of the animals were determined. As expected, mice of the HFD group had significantly increased body and liver weights, whereas the values of the CD and SD group were at the lower limit of the age- and sex-specific normal range [33]. The reason for the increased body and liver weights is diet-induced fat accumulation, examined by analysis of liver fat content as well as determination of hepatic cholesterol. Mice of the HFD group showed significantly increased liver fat content of more than 10%, whereas both the controls were in the same range of liver fat content as healthy mice, which is about 5–8% [24,34].

Contrary to this finding, the hepatic cholesterol assay revealed a significantly increased cholesterol content in the CD group. Cholesterol has lipotoxic effects, mainly mediated by the induction of oxidative stress, which are able to activate pro-inflammatory signaling pathways and thus lead to NAFLD progression, even in lean individuals [35]. This provides a possible explanation for macroscopically observed pathological changes in the livers of the CD group. Therefore, in our experiments, the liver of the CD group with normal liver fat-content but high cholesterol showed a liver LGI equal to mice fed with HFD, which is comparable to previous results of HFD vs. carbohydrate-rich diet [36]. We suggest that starch is the main mediator of the elevated cholesterol content and the pro-inflammatory effects seen in the CD group, which is in line with results of Duwaerts et al. [37], describing a pathogenic effect of diets rich in starch on the liver, independent of calories and nutrient proportions [37]. From a nutritional point of view, CD as well as HFD are rich in lards, contributing to a pro-inflammatory profile [38] in both groups. Additionally, a high carbohydrate amount in the CD also contributes to that [20,21], whilst an elevated protein content, such as in the SD group, exerts more anti-inflammatory effects [22].

To explore the dietary effect on the mice livers in depth, they were histologically processed and analyzed according to aspects that are relevant in NAFLD diagnostics [23,24]. The NAS, created within the scope of evaluation, showed a more than fourfold increase in the HFD group compared to the CD and SD group, mainly due to steatosis. In the HFD group, this indicates the beginning and progression of NAFLD, described in the literature as an organic manifestation of the metabolic syndrome [5]. This damaging effect on the livers also became apparent by significantly increasing ALT and AST plasma values in the HFD group (Supplementary Materials Table S1). A persistent LGI may be a mechanistic link between obesity and the various comorbidities, such as NAFLD. There is strong evidence from the literature that obesity exerts an influence on the immune system and manifests itself as a condition of chronic LGI [11,39]. LGI is triggered or promoted by intrinsic stress factors, tissue dysfunction and changes in homeostatic checkpoints, which commonly occur in obesity [7]. The expression and characterization of a liver manifestation of LGI, triggered by HFD-induced obesity, were therefore another focus of the experiments.

Further characterization of obesity-associated LGI in the liver focused on the local cellular immune response. Cell damage induced in the context of NAFLD can lead to reduced liver function [40] with extensive consequences on the hepatic synthesis performance. In addition to inflammatory foci in the liver, assessed by NAFLD scoring, the number of granulocytes and the number of macrophages was determined. Due to the heterogeneity of the macrophage population in the liver, we only counted F4/80+ cells, mainly consisting of tissue resident Kupffer cells and macrophages recruited from the bloodstream [41]. In the calculated ratios of both granulocytes and macrophages to the number of hepatocytes, significantly increased values were observed in the HFD group as well as in the CD group. Not only mature macrophages were attracted from the bloodstream, but also myeloid progenitor cells through a closely regulated interaction of genes for chemokines, chemokine receptors, adhesion molecules, myeloid markers and inflammatory cytokines [42]. Therefore, as a further step to characterize diet induced liver LGI, hepatic expression of different cytokines was determined.

In addition to hepatocytes themselves, it is commonly known that immune cells in the liver also play a major role in the expression inflammatory mediators [43]. In our work, determined hepatic expression levels of cytokines do not provide a homogeneous pattern. According to TNFα elevations in obesity shown by other working groups [10,39,44], we were able to show an elevation of hepatic TNFα expression in the HFD group too, but also in the CD group compared to the SD group. Whilst the expression of pro-inflammatory TNFα was in line with the shown cellular immune response, the results of the other three analyzed cytokines differed and were partially contradictory. In contrast to increased IL-6 levels in obesity described in the literature [45], the HFD group only showed a slight increase in hepatic expression compared to the SD group. The hepatic IL-6 expression in the CD group was even higher than in the HFD group, again underpinning the pro-inflammatory potential of the carbohydrate-rich CD. A significantly lower IL-1β and elevated IL-10 level in the CD group may be explainable by an overshooting anti-inflammatory counter-regulation, as IL-10 especially is able to alter the expression of other cytokines [46].

In conclusion, our results show obesity derived liver damage associated with an organic manifestation of a LGI in the livers of the HFD group. Even if not leading to a significant increase of NAS in the CD group over the experimental time, an increased inflammatory potential was shown in the group fed with a diet rich in carbohydrates. Therefore, when studying diet-induced obesity with a focus on inflammatory processes like LGI, we would not suggest using a control diet disproportionately rich in carbohydrates.

## Figures and Tables

**Table 1:** Primers used for quantitative real-time PCR.

Primer	Orientation	Sequence
RPS18	Forward Reverse	5′-AGGATGTGAAGGATGGGAAG-3′ 5′-TTGGATACACCCACAGTTCG-3′
TNFα	Forward Reverse	5′-ACATTCGAGGCTCCAGTGAATTCGG-3′ 5′-GGCAGGTCTACTTTGGAGTCATTGC-3′
IL-1β	Forward Reverse	5′-CCCAAGCAATACCCAAAGAA-3′ 5′-TTGTGAGGTGCTGATGTACCA-3′
IL-6	Forward Reverse	5′-TCTGACCACAGTGAGGAATGTCCAC-3′ 5′-TGGAGTCACAGAAGGAGTGGCTAAG-3′
IL-10	Forward Reverse	5′-GCCTTGCAGAAAAGAGAGCT-3′ 5′-AAAGAAAGTCTTCACCTGGC-3′

## Supplementary Materials

The following are available online at https://www.mdpi.com/2227-9059/8/12/587/s1, Table S1: Blood Parameters blood glucose concentration, triglyceride concentration, AST and ALT concentration in the high fat diet (HFD), control diet (CD) and standard diet (SD) group presented as mean ± standard deviation, ImageJ code S1: Code for semiautomatic quantification of liver fat, ImageJ code S2: Code for quantification of F4/80+-cells, Figure S1: Representative images for scoring of steatosis (Score 0-3, 50 x magnification, scale bar represents 200 µm valid for all four), ballooning (Score 0-1, 100x magnification, scale bar represents 100 µm valid for both) with black arrows indicating damaged cells, and inflammation (Score 0-2, 200x magnification, scale bar represents 50 µm valid for all three) with black arrows indicating inflammatory foci
