# Supplementary material for: Dietary-Induced Low-Grade Inflammation in the Liver
Source: Biomedicines. 2020 Dec 9;8(12):587. doi: 10.3390/biomedicines8120587 (PMC7763065; doi:10.3390/biomedicines8120587)
Supplement: Supplementary file 1 [file biomedicines-08-00587-s001.pdf]

Supplementary Materials (in order of appearance in the main text)**:**

Table S1: Blood Parameters blood glucose concentration, triglyceride concentration, AST and ALT concentration in the different groups presented as mean ± SD

Parameter	HFD	CD	SD	Significances
Blood Glucose [mmol/L]	13.72 ± 1.93	12.10 ± 2.30	11.49 ± 2.10	HFD vs. CD P=0.0447<br>HFD vs. SD P=0.0037<br>CD vs. SD P>0.05
Triglycerides [mg/dL]	63.32 ± 13.56	51.23 ± 13.11	58.12 ± 24.52	HFD vs. CD P=0.0227<br>HFD vs. SD P>0.05<br>CD vs. SD P>0.05
ALT [U/L]	75.21 ± 46.52	21.73 ± 10.12	27.48 ± 7.14	HFD vs. CD P<0.0001<br>HFD vs. SD P<0.0001<br>CD vs. SD P>0.05
AST [U/L]	120.30 ± 51.41	71.16 ± 23.60	65.71 ± 7.79	HFD vs. CD P<0.0001<br>HFD vs. SD P=0.0004<br>CD vs. SD P>0.05

ImageJ code S1. Code for quantification of liver fat:

```
run("Set Scale...", "distance=285 known=50 pixel=1 unit=µm global");
run("Subtract Background...", "rolling=50 light");
run("8-bit");
run("Brightness/Contrast...");
setMinAndMax(30,225);
run("Apply LUT");
run("Enhance Contrast...", "saturated=0.4 normalize");
run("Sharpen");
run("Threshold...");
setAutoThreshold("Default dark");
setThreshold(185, 255);
setOption("BlackBackground", false);
run("Convert to Mask");
run("Close");
run("Remove Outliers...", "radius=7 threshold=50 which=Dark");
run("Fill Holes");
run("Analyze Particles...", "size=2-600 circularity=0.25-1.00 show=[Overlay Masks] display summarize add");
roiManager("Show All with labels");
roiManager("Show All");
close();
```

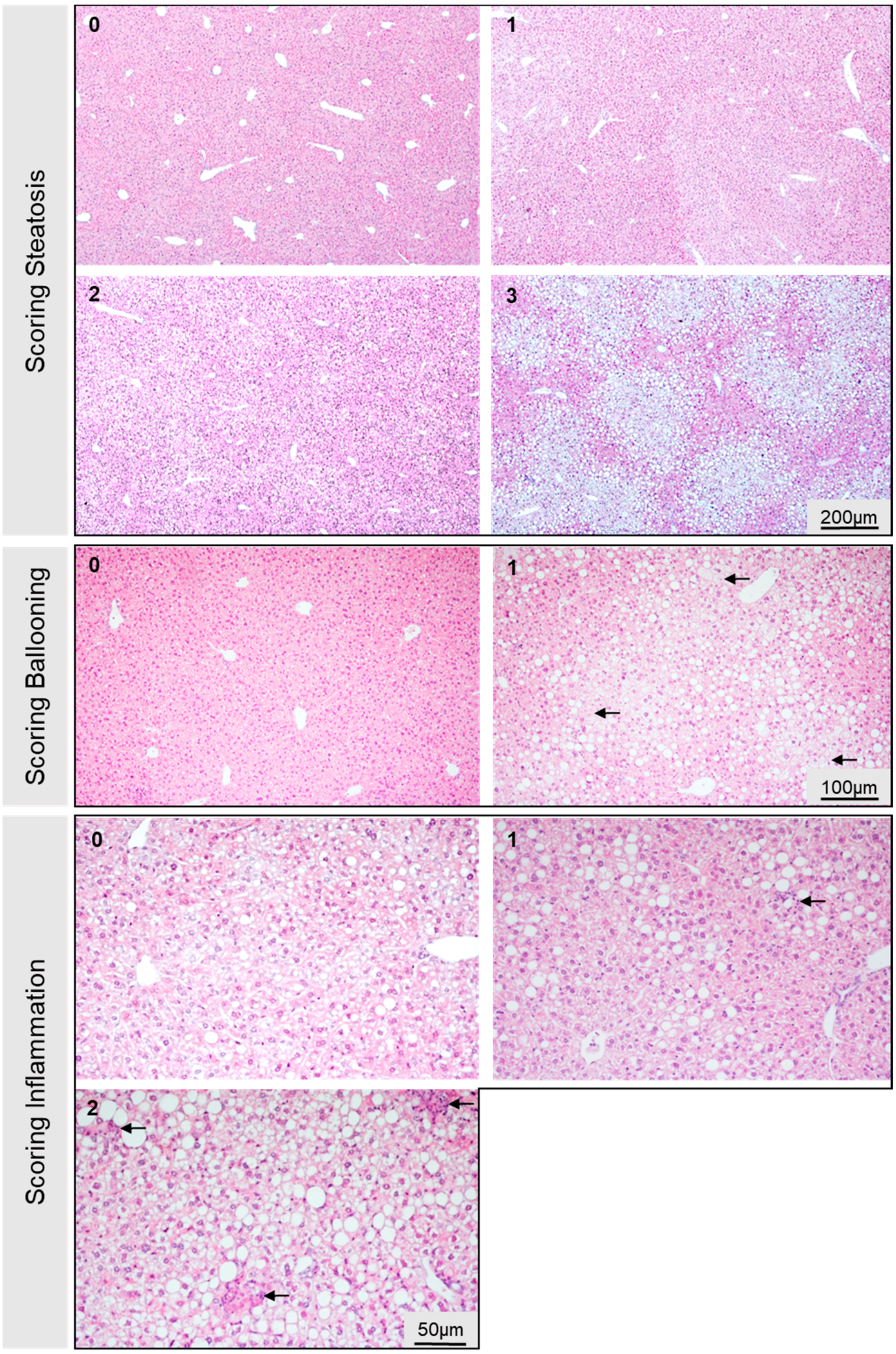

Figure S1. Representative images for scoring of steatosis (Score 0-3, 50 x magnification, scale bar represents 200 µm valid for all four), ballooning (Score 0-1, 100x magnification, scale bar represents 100 µm valid for both) with black arrows indicating damaged cells, and inflammation (Score 0-2, 200x magnification, scale bar represents 50 µm valid for all three) with black arrows indicating inflammatory foci.

ImageJ code S2. Code for quantification of F4/80^{+}-cells:

```
// Color Thresholder 1.46r
// Autogenerated macro, single images only!
min=newArray(3);
max=newArray(3);
filter=newArray(3);
a=getTitle();
run("HSB Stack");
run("Convert Stack to Images");
selectWindow("Hue");
rename("0");
selectWindow("Saturation");
rename("1");
selectWindow("Brightness");
rename("2");
min[0]=0;
max[0]=218;
filter[0]="pass";
min[1]=0;
max[1]=255;
filter[1]="pass";
min[2]=0;
max[2]=255;
filter[2]="pass";
for (i=0;i<3;i++){
  selectWindow(""+i);
  setThreshold(min[i], max[i]);
  run("Convert to Mask");
  if (filter[i]=="stop")  run("Invert");
}
imageCalculator("AND create", "0","1");
imageCalculator("AND create", "Result of 0","2");
for (i=0;i<3;i++){
  selectWindow(""+i);
  close();
}
selectWindow("Result of 0");
close();
selectWindow("Result of Result of 0");
rename(a);
// Colour Thresholding-------------
run("Invert");
run("Make Binary");
run("Fill Holes");
run("Analyze Particles...", "size=0.125-2.00 show=Outlines display exclude clear include summarize add slice");
```
